# The Role of Edaphic Environment and Climate in Structuring Phylogenetic Pattern in Seasonally Dry Tropical Plant Communities

**DOI:** 10.1371/journal.pone.0119166

**Published:** 2015-03-23

**Authors:** Marcelo Freire Moro, Igor Aurélio Silva, Francisca Soares de Araújo, Eimear Nic Lughadha, Thomas R. Meagher, Fernando Roberto Martins

**Affiliations:** 1 State University of Campinas—UNICAMP, Departamento de Biologia Vegetal, Bloco M, CEP 13.083–970, Campinas, São Paulo, Brazil; 2 Federal University of Ceará—UFC, Departamento de Biologia, Centro de Ciências, Universidade Federal do Ceará, Campus do Pici, Bloco 906, CEP 60455–760, Fortaleza, CE, Brazil; 3 Royal Botanic Gardens, Kew, Richmond, Surrey, TW9 3AB, United Kingdom; 4 School of Biology, University of St Andrews, St Andrews, Fife, KY16 9TH, United Kingdom; USDA-Agricultural Research Service, UNITED STATES

## Abstract

Seasonally dry tropical plant formations (SDTF) are likely to exhibit phylogenetic clustering owing to niche conservatism driven by a strong environmental filter (water stress), but heterogeneous edaphic environments and life histories may result in heterogeneity in degree of phylogenetic clustering. We investigated phylogenetic patterns across ecological gradients related to water availability (edaphic environment and climate) in the Caatinga, a SDTF in Brazil. Caatinga is characterized by semiarid climate and three distinct edaphic environments – sedimentary, crystalline, and inselberg –representing a decreasing gradient in soil water availability. We used two measures of phylogenetic diversity: Net Relatedness Index based on the entire phylogeny among species present in a site, reflecting long-term diversification; and Nearest Taxon Index based on the tips of the phylogeny, reflecting more recent diversification. We also evaluated woody species in contrast to herbaceous species. The main climatic variable influencing phylogenetic pattern was precipitation in the driest quarter, particularly for herbaceous species, suggesting that environmental filtering related to minimal periods of precipitation is an important driver of Caatinga biodiversity, as one might expect for a SDTF. Woody species tended to show phylogenetic clustering whereas herbaceous species tended towards phylogenetic overdispersion. We also found phylogenetic clustering in two edaphic environments (sedimentary and crystalline) in contrast to phylogenetic overdispersion in the third (inselberg). We conclude that while niche conservatism is evident in phylogenetic clustering in the Caatinga, this is not a universal pattern likely due to heterogeneity in the degree of realized environmental filtering across edaphic environments. Thus, SDTF, in spite of a strong shared environmental filter, are potentially heterogeneous in phylogenetic structuring. Our results support the need for scientifically informed conservation strategies in the Caatinga and other SDTF regions that have not previously been prioritized for conservation in order to take into account this heterogeneity.

## Introduction

Plant distributions and assemblages are an effective representation of impacts of environmental heterogeneity and change. For example, ecological modeling of plant distributions is potentially informative about the biological impacts of recent climate change [1–3]. The relationship between plants and edaphic conditions and the heterogeneity of growth forms that occur in plants lineages introduce different time-frames for response. Climatic conditions are likely to change more rapidly over time than soil, and short-lived herbaceous species might have a more rapid local species turnover than long-lived woody plants. Also, short-lived herbaceous species might be expected to adapt more rapidly than slower-growing woody species, owing to life-history differences [4]. Thus, heterogeneity in plant assemblages is likely to reflect a variety of environmental drivers at different spatial or temporal timescales.

Seasonally dry tropical plant formations (SDTF) are an important component of tropical vegetation and one of the most threatened biomes in the world [5–7]. SDTFs may be broadly defined as formations that occur in tropical regions characterized by pronounced seasonality in rainfall distribution, resulting in several months of drought (a period of at least 5 months receiving less than 100 mm [8,9]). The physiognomies shown by SDTF are heterogeneous, including formations ranging from tall forests to short cactus scrub, but is mostly tree-dominated and semi-deciduous to deciduous during the dry season [5,8]. The most extensive contiguous areas of SDTF are in the neotropics, comprising more than 60% of the remaining global stands of this vegetation [7]. Neotropical SDTFs experience a high deforestation rate (12% between 1980 and 2000), highlighting an urgent priority for conservation [7]. SDTFs are important ecosystems that are being rapidly degraded. It is critically important that we obtain a better understanding of the ecological dynamics of this vegetation type so that it can be better managed in the future.

A clear understanding of the manner in which vegetation patterns in SDTFs vary in relation to environmental heterogeneity can make an important contribution to understanding adaptation to specific environmental conditions (environmental filtering) over different temporal and spatial scales (e.g. climate versus edaphic environment), and different time-scales for species turnover or adaptation (e.g. owing to life history differences between woody and non woody species). An understanding of such properties of local species assemblages and concomitant impact on local biodiversity is critical for informing conservation strategy.

One of the largest areas of SDTF is the Caatinga (over 800,000 km^2^) in northeast Brazil [7,10]. Originally occupying 11% of the Brazilian territory, the Caatinga occurs predominantly in lowland crystalline terrain at altitudes below than 500 m. Some Caatinga areas also occur in terrains of sedimentary origin, which offer very different edaphic conditions when compared with crystalline sites. Soils on crystalline terrains are richer in nutrients; however, they are shallow and stony, retaining water only for a short period after the rainy season [10]. Conversely, soils on sedimentary terrains are usually poor in nutrients; but they are deeper, usually retaining water for a longer period after the end of rainy season [10–13]. Thus, plants in sedimentary soils are expected to have a better water supply in the dry season than those on crystalline substrates, but fewer nutrients for their growth [10,13]. In addition, scattered across the semi-arid Caatinga, there are many inselbergs (rocky outcrops) where the rocky basement emerges, and soils are consequently very shallow or absent, with even less water retention that crystalline soils. Sedimentary, crystalline and inselberg environments have been shown to contain different sets of species within Caatinga [12,14–17].

The majority of plant diversity studies in Caatinga have focused on traditional measures of diversity, such as number of species and the Shannon index [18,19]. However, the increasing availability of molecular phylogenies has fuelled growing interest in using phylogenetic approaches to study drivers that influence patterns of plant diversity [20]. Phylogenetic diversity is more inclusive than a simple count of species or types, since it quantifies the evolutionary history of species [21–23]. In addition, conservation biologists are frequently interested in preserving phylogenetic diversity of communities because it is fundamental to maximizing evolutionary options for the future [22,24,25].

The Caatinga represents a good experimental system for investigating patterns of phylogenetic diversity, because there are clear spatial and temporal dimensions that can be explored. There is growing evidence that the Caatinga exhibits relatively high β-diversity in comparison with other adjacent biomes of comparable area [26], and this has been attributed to the interaction between spatial environmental heterogeneity and temporal scales of ecological and evolutionary response. There is a clear need to investigate this interaction further.

Plants in Caatinga are mainly limited by water availability, as the annual rainfall is concentrated in just three or four consecutive months [10,27]. A consequence of annual drought is that most plants growing in these semi-arid regions have developed means to avoid or tolerate water loss [28]. For instance, some plants avoid water-deficit stress by undergoing the dry periods as drought-tolerant seeds (therophytes *sensu* Raunkiaer [29]). Succulents and cacti, in contrast, avoid water-deficit stress by storing water in modified tissues (stems or leaves) during periods of water scarcity, whereas drought tolerant species are capable of surviving water loss without suffering irreparable damage to vegetative tissues [28]. If these ecological strategies are conserved in the evolution of plant lineages (i.e., closely related species share similar ecological strategies [30]), the extended drought in the Caatinga can be expected to assemble closely related species [20,21,23], resulting in phylogenetic clustering. Indeed, niche conservatism may be a general property of SDTFs [9], potentially leading to phylogenetic clustering; and it has been suggested that Caatinga communities may show more phylogenetic niche conservatism than other South American biomes that occur across a similar spatial scale [26].

**Fig 1:**
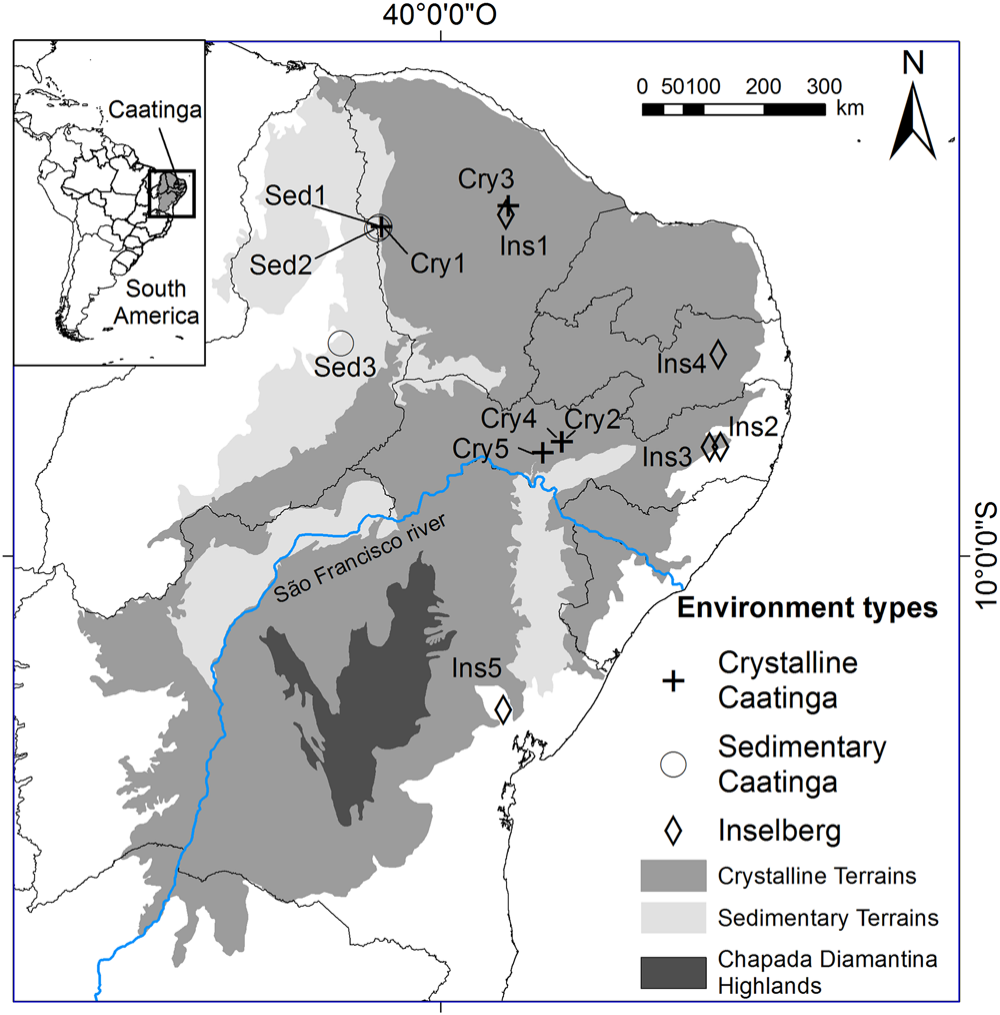
Geographical location of the floristic studies that also reported Raunkiaerian life-forms for the species in Caatinga and that were analyzed in this study.

In order to evaluate the impacts of different environmental drivers on patterns of phylogenetic diversity, we analyzed the role of edaphic environment and climate in determining the phylogenetic pattern of plant species of Caatinga sites. To test whether increasing annual temperature, decreasing annual rainfall and shallow soils promote phylogenetic clustering of plant assemblages, we assessed 13 sites across the entire Caatinga domain (Fig. 1). In addition, we evaluated the extent to which edaphic environment and climate differentially modify phylogenetic pattern of woody and herbaceous species, since those two groups of species are expected to show different rates of species turnover and evolutionary adaptation [4] and also show different ecological strategies to deal with water loss, i.e. woody species tolerate whereas herbaceous species avoid [28]. Thus, we investigated the importance of environmental filtering owing to water availability in shaping phylogenetic diversity in Caatinga. (1) What is the relationship between edaphic environment and the phylogenetic community structure? (2) How do climatic factors that influence water availability influence phylogenetic community structure? In addition, we investigated the extent to which response to such environmental drivers takes place over different timescales for adaptation through the following questions. (3) Is phylogenetic clustering equally prevalent in woody species and in herbaceous species? (4) Does more recent phylogenetic diversification result in phylogenetic clustering along different environmental axes than longer term phylogenetic diversification; and if so, how might these relate to environmental factors that might show different spatial and temporal patterns (e.g. edaphic environment versus climate)?

## Materials and Methods

### Ecological data

We compiled data from 13 floristic surveys in the Caatinga Phytogeographical Domain, NE Brazil, with information on Raunkiaerian life forms of species (Table 1; Fig. 1). These studies were selected from a thorough literature survey of plant diversity on the Caatinga Phytogeographical Domain that identified 131 surveys with site-based floristic or phytosociological information [17]. From this larger dataset we selected all lists that sampled the general flora of each site (i.e. those that included plants of all habits, from woody to herbaceous species) and that included Raunkiaer’s life-form for each species. We then created a database of all species reported in these studies, excluding exotic species and species assigned only to genus or family level. We assumed that species with a cf. status (i.e. species identified with a certain degree of uncertainty) were correctly identified. Ferns and Lycophytes were also excluded from the analysis, because they represent very old clades, which would bias the phylogenetic metrics, and are a species-poor component in Caatinga.

**Table 1:** Studies presenting floristic lists (associated with Raunkiaer’s life-forms data) used in our analyses.

Municipality, State	Substrate	Reference	Latitude	Longitude
Crateús, CE	crystalline	Araújo *et al*. (2011) [13]	-5.133	-40.866
Floresta, PE	crystalline	Costa *et al*. (2009) [50]	-8.312	-38.195
Quixadá, CE	crystalline	Costa *et al*. (2007) [51]	-4.826	-38.969
Floresta, PE	crystalline	Rodal *et al*. (2005) [52]	-8.309	-38.202
Floresta, PE	crystalline	Rodal *et al*. (2005) [52]	-8.476	-38.480
Crateús, CE	sedimentary	Araújo *et al*. (2011) [13]	-5.167	-40.933
Crateús, CE	sedimentary	Araújo *et al*. (2011) [13]	-5.133	-40.900
São José do Piauí, PI	sedimentary	Mendes & Castro (2010) [53]	-6.854	-41.471
Quixadá, CE	inselberg	Araújo *et al*. (2008) [54]	-4.956	-39.024
São Joaquim do Monte, PE	inselberg	Gomes & Alves (2010) [55]	-8.382	-35.844
Agrestina, PE	inselberg	Gomes & Alves (2010) [55]	-8.391	-36.010
Esperança, PB	inselberg	Porto *et al*. (2008) [56]	-7.017	-35.881
Feira de Santana, BA	inselberg	França *et al*. (2005) [57]	-12.272	-39.061

We classified all reported life forms according to the five main Raunkiaer [29] categories: (1) phanerophytes, which have buds that are well above the ground during the dry season; (2) chamaephytes, which have buds close to the ground; (3) hemicryptophytes, which have buds at the ground level; (4) cryptophytes, which have buds below ground; and (5) therophytes, which are annual plants that complete their life-cycle, reproduce and die during a single rainy season. We used Raunkiaer categories because they are based on life history features that are closely aligned with adaptation to the ecological conditions highlighted in our study. When one of the studies reported a life-form using a category different from those originally proposed by Raunkiaer (e.g. succulent and climbers), we reclassified species back into one of the Raunkiaerian categories. Aerophytes, epiphytes and hemiparasites were reclassified as phanerophytes. Cacti and succulents were reclassified as chamaephytes or phanerophytes depending on the size of adult plants. Climbers were reclassified as phanerophytes, chamaephytes or therophytes, depending on their senescence in the dry season. In these studies, a few species were not classified into any Raunkiaer category. In these cases, we assigned species to Raunkiaer categories based on personal field experience or consultation with other taxonomic specialists. When more than one life form was associated with a single species (e.g. a species was reported as chamaephyte in one site, but phanerophyte in another), we considered that the life form with buds less protected was applicable for the species (in this case, a phanerophyte).

Because the Caatinga is a semi-arid region, we considered climatic variables related to temperature and precipitation as potentially significant ecological drivers of species distributions. We obtained five climatic variables for each site from the global climate model WorldClim [31] using DIVA GIS 7.3 software [32]: annual mean temperature, annual precipitation, precipitation seasonality, precipitation of the wettest quarter, and precipitation of the driest quarter. In order to take into account variation in edaphic environments on species distributions, we classified each site as sedimentary, crystalline, or inselberg (Table 1; Fig. 1), depending on the type of edaphic environment reported by the authors of the study.

### Phylogenetic data

We obtained a phylogenetic tree for all plant species in our database using the online mega-tree Phylomatic, a phylogenetic database and software toolkit for the assembly of phylogenetic trees [33]. Phylogenetic relationships among species from different families were estimated from the current Phylomatic tree (R20100701). The backbone of the Phylomatic tree is the phylogenetic relationships among Angiosperm Phylogeny Group orders [34]. Phylomatic generates a supertree assembled by hand, rather than by an automated supertree algorithm, and conflicting branching patterns were resolved subjectively. Phylomatic outputs are intended to represent a pragmatic approximation of the true phylogeny of seed plants [33]. Branch lengths were based on minimum ages of nodes determined for families and higher orders from fossil data [35]. We placed undated nodes in the tree evenly between dated nodes with the branch length adjustment algorithm in Phylocom [36]. This algorithm took the phylogeny generated by Phylomatic, fixed the root node at 137 million years before present (i.e., the age of the eudicots clade) and fixed other nodes for which we had age estimates from Wikström *et al*. [35]. Phylomatic then sets all other branch lengths by placing the nodes evenly between dated nodes, and between dated nodes and terminals [36]. This has the effect of minimizing variance in branch length, within the constraints of dated nodes. Phylomatic thus produces a pseudo-chronogram that can be useful for estimating phylogenetic distance (in units of time) between taxa for analysis of phylogenetic community structure.

### Phylogenetic diversity

We calculated two measures of phylogenetic diversity—the mean phylogenetic distance (MPD) and the mean nearest neighbor phylogenetic taxon distance (MNTD)—for each Caatinga site. MPD is defined as the mean phylogenetic distance among all pairwise combinations of species, and MNTD is defined as the mean phylogenetic distance to the nearest relative for all species in a sample [37,38]. Thus, MPD is a measure of tree-wide phylogenetic distance of species (deeper, older phylogenetic relationships), and MNTD is a measure of terminal (branch tip) phylogenetic association of species (i.e., with congeners or confamilials; [37]). These two measures of phylogenetic diversity capture different time-frames of ecological association and evolutionary adaptation, with MPD representing longer term ecological association and also potentially a longer time frame for adaptation.

We calculated MPD and MNTD over all species from each site (general flora) as well as obtaining separate estimates for woody (phanerophytes) and herbaceous (therophytes, cryptophytes, hemicryptophytes, and chamaephytes) species (S1 Dataset). The magnitudes of estimates of MPD and MNTD are known to be sensitive to species number [36]. Because we are making comparisons across habitats and life forms that differ in number of species, for subsequent analyses we calculated the standardized effect size (SES) of MPD and MNTD, in which the original diversity measures are standardized against null communities generated by randomization as follows: SES = (observed value—null value) / sd null value, based on the observed value of the metric, the mean metric value of null communities, and the standard deviation of measure estimates from 1,000 random permutations of the data. In the case of our analysis, we generated random values by reshuffling taxa labels across the tips of the phylogenetic tree of all species sampled. From SES calculated for MPD and MNTD, multiplying by -1 yields scores known respectively as the Net Relatedness Index (NRI) and the Nearest-Taxon Index (NTI), respectively [20,39]. Positive values of NRI or NTI indicate that the taxa present at a site are more closely related to each other than expected by chance (phylogenetic clustering) whereas negative values indicate that taxa are more evenly distributed across the phylogeny than expected by chance (phylogenetic overdispersion) [20,21,23]. Because standardized effect sizes are scaled in units of standard deviation, values of NRI or NTI > 1.96 indicate statistically significant phylogenetic clustering while values < -1.96 indicate statistically significant phylogenetic overdispersion.

### Phylogenetic signal in life forms

To test the assumption of niche conservatism across taxa, we estimated the phylogenetic signal associated with life forms, i.e. trait similarity among species associated with phylogenetic relatedness [30], based on the minimum number of character state changes across the tree involving life form [40]. We considered each life form category as a character state. If related species have similar life forms, the number of character state changes will be lower than expected at random [40]. The minimum number of changes was compared to a distribution of 999 random numbers of changes, which were obtained by swapping the trait states across the tips of the tree [40].

### Data analysis

#### Selection of climate variables

We tested for multicollinearity among the five climate variables using variance inflation factors (VIF), which measure the proportion by which the variance of a regression coefficient is inflated in the presence of other explanatory variables [41]. We tested the multicollinearity with multiple regressions considering the NRI as the dependent variable. As a result of this test, we excluded from subsequent ANCOVA analyses two climate variables: precipitation of the wettest quarter and precipitation seasonality.

Spatial autocorrelation of independent variables violates the assumption of data independence [42]. Therefore, we tested for autocorrelation among our independent climate variables (annual precipitation, annual mean temperature, and precipitation during the driest quarter) by calculating Moran’s I for a series of 6 distance classes scaled against the largest distance between sites, resulting in spatial limits of approximately 200 km. Positive values of Moran’s I indicate positive autocorrelation (spatial aggregation) and negative values indicate negative autocorrelation (spatial over-dispersion) [43].

#### ANCOVAs

Since we found spatial autocorrelation in climate variables (Table 2), we incorporated the spatial structure of the data into the ANCOVA. We used an approach that has been called eigenvector-based spatial filtering or the ‘principal coordinate of neighbor matrices’ (PCNM), which extracts eigenvectors from a connectivity matrix expressing the spatial relationship among plots [44,45]. These eigenvectors (i.e. the spatial filters) express the relationships among plots at decreasing spatial scales in such a way that the first eigenvectors (those related to large eigenvalues) tend to describe broad-scale spatial patterns, whereas eigenvectors with small eigenvalues tend to describe local patterns (the spatial structure of the regression; [44,45]). Eigenvalues were therefore used as additional predictors of the response variables in the minimum adequate model in an attempt to reduce the autocorrelation in the residuals [45]. In our ANCOVA, we used the spatial filter associated with the first eigenvalue as that was substantially larger in magnitude than the remaining eigenvalues.

**Table 2:** Tests for spatial autocorrelation of phylogenetic diversity measures and climate variables in Caatinga, Brazil.

	Dependent variables (phylogenetic diversity)												Independent variables (climate)					
Distance centroid (km)	NRI_all_	P	NRI_wood_	P	NRI_herb_	P	NTI_all_	P	NTI_wood_	P	NTI_herb_	P	Annual Mean Temp.	P	Annual Mean Precip.	P	Precip. of the Driest Quarter	P
101	**0.92**	**0.001**	**0.69**	**0.001**	**0.60**	**0.001**	**0.62**	**0.001**	0.21	0.14	**0.36**	**0.03**	**0.60**	**0.001**	**0.73**	**0.001**	**0.29**	**0.05**
249	0.09	0.40	0.17	0.22	-0.45	0.08	-0.35	0.20	-0.22	0.51	-0.36	0.18	0.12	0.33	0.28	0.09	0.01	0.62
350	0.04	0.42	-0.03	0.73	0.01	0.56	0.16	0.12	-0.03	0.77	-0.11	0.87	0.02	0.51	-0.29	0.19	0.24	0.10
438	-0.00	0.60	-0.05	0.85	0.13	0.18	-0.05	0.83	-0.26	0.32	0.06	0.38	-0.16	0.62	**-1.28**	**0.000**	-0.18	0.62
552	**-0.58**	**0.03**	-0.13	0.82	**-0.68**	**0.007**	-0.15	0.76	0.12	0.33	-0.32	0.27	-0.98	0.001	-0.07	0.49	-0.03	0.76
735	**-0.99**	**0.001**	**-1.09**	**0.001**	-0.04	0.84	**-0.75**	**0.001**	-0.39	0.12	-0.14	0.79	-0.07	0.96	-0.10	0.36	**-0.88**	**0.001**

The values of Moran’s I coefficient are shown for each distance class, calculated with equal number of pairs for each distance class. Significant spatial autocorrelation values at α = 0.05 are in bold. Numbers are presented to two decimal places or two significant digits.

Finally, we performed ANCOVAs to determine whether variation in annual mean temperature, annual mean precipitation, and precipitation of the driest quarter was related to variation in NRI and NTI, considering edaphic environment as a covariate [46]. As noted above, we also used the first spatial filter as an additional covariate in the analyses.

#### Partitioning the variation of the phylogenetic diversity

We decomposed the variation of phylogenetic diversity between climate and edaphic environment components to evaluate their relative roles in constraining the plant community structure. We followed the procedure described in Legendre & Legendre [43] to calculate the portion of the variation of each diversity measure (**Y**) that is attributed to climate variables (**X**) and to edaphic environment (**W**). We first did an ANCOVA, regressing **Y** against **X** and **W**. The resulting value of R^2^ determined the portion of variation [a + b + c] related to climate [a], to soil type [c], and to both variables [b]. We did a multiple regression of **Y** against **X**, from which the resulting value of R^2^ determined [a + b]. We did a linear regression of **Y** against **W**, from which the resulting value of R^2^ determines [b + c]. Then, the portion [b] was obtained by the equation [b] = [a + b] + [b + c]–[a + b + c] [43].

We did phylogenetic analyses using R software [47], and ANCOVAs and multiple regressions using SAS for Windows 9.4 (SAS Institute Inc., Cary, NC, USA). We tested for phylogenetic signal in life forms with the ‘phylo.signal.disc’ function, which was developed *ad hoc* by E.L. Rezende (pers. comm.) and corresponds to the ‘fixed tree, character randomly reshuffled model’ proposed by Maddison and Slatkin [40]. We calculated MPD, MNTD, and their respective SES using the ‘picante’ package (version 0.2–0; [48]). All calculations for the spatial analyses (i.e. Moran’s I and spatial filters) were conducted in SAM version 4.0 [49].

## Results

### Niche conservatism across taxa

Five out of the 13 surveys that we selected from the literature were in crystalline soil, three in sedimentary soil, and five in inselbergs (Table 1; Fig. 1; [13,50–57]). Overall, these studies reported 752 plant species: 484 woody (shrubs and trees) and 268 herbaceous (non-woody or subshrubs). We found significant phylogenetic signal in the life forms of Caatinga plants. The observed number of character state changes across the phylogenetic tree was lower than expected by chance (P < 0.001), indicating overall phylogenetic niche conservatism. The median of random trait state changes was 373 (minimum and maximum 349 and 388, respectively), whereas the observed number of character state changes was 329.

### Spatial autocorrelation

The climate variables showed positive spatial autocorrelation in shorter distance classes, but this was significant only in the shortest distance class (with distance centroid of 101km) (Table 2). Negative spatial autocorrelation was evident in the climate variables over all the longer distance classes (with distance centroid of 438km or more) but the distance classes in which the negative values were significant differed between climate variables (Table 2). Spatial autocorrelation was also evident in NRI and NTI values. Significant, positive values of Moran’s I indicated spatial autocorrelation in all estimates of NRI in the shortest distance class (NRI_all_, NRI_wood_ and NRI_herb_). Significant positive autocorrelation was also seen in NTI_all_ and NTI_herb_ in the shortest distance class, while for NTI_wood_ the equivalent value was also positive but not significantly so. Negative values of Moran’s I were prevalent in the longer distance classes (with distance centroid of 438km or more) and significantly so for NRI_all_ and NTI_all_ at the longest distance class. NRI_wood_ also showed significant negative autocorrelation at the longest distance class, as did NRI_herb_ at the second-longest distance class but equivalent values for NTI_wood_ and NTI_herb_ were not significant. To correct the spatial autocorrelation in the data we extracted with PCNM analysis one significant spatial filter to be used in the ANCOVA.

### Phylogenetic diversity

**Fig 2:**
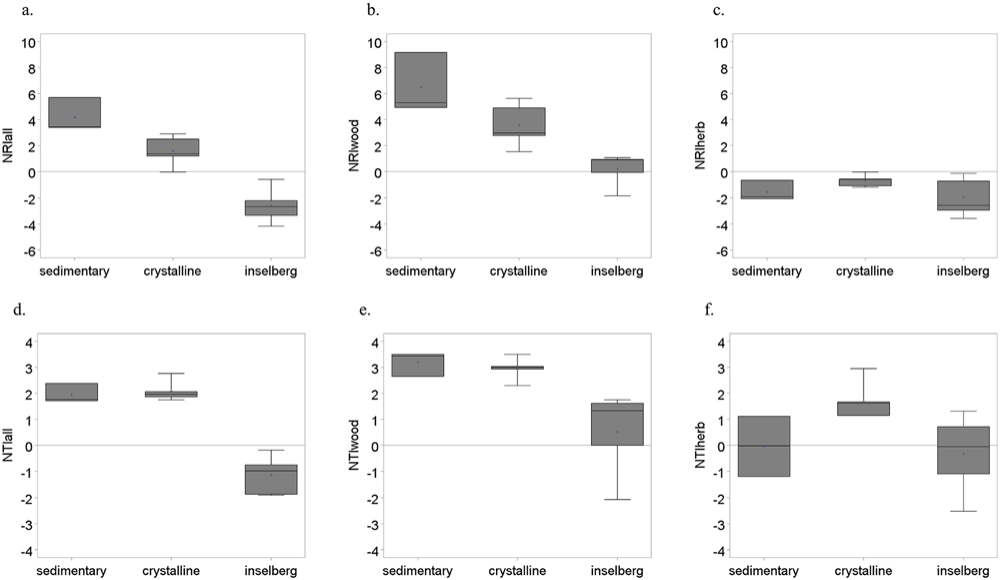
Phylogenetic diversity of Caatinga plants in different edaphic environments (sedimentary, crystalline, inselberg), NE Brazil: net relatedness index for (a) all species (NRI_all_), (b) woody species (NRI_wood_), and (c) herbaceous species (NRI_herb_); nearest taxon index for (d) all species (NTI_all_), (e) woody species (NTI_wood_), and (f) herbaceous species (NTI_herb_).

When all species are considered, phylogenetic overdispersion as measured by NRI_all_ and NTI_all_ was evident for inselbergs (Fig. 2a,d), reflected in negative values of NRI_all_ and NTI_all_ for each individual inselberg site (Tables 3 and 4), though only NRI_all_ values were significant. This pattern of phylogenetic overdispersion in inselbergs, in contrast to phylogenetic clustering in crystalline and sedimentary sites, was corroborated by the ANCOVAs (Tables 5 and 6). The climate variables analyzed showed no significant effect on either NRI_all_ or NTI_all_ (Tables 5 and 6). Partitioning of variation explained solely by climate variables and edaphic environment was similar for NRI_all_ and NTI_all_ with the edaphic environment explaining variation of NRI_all_ and NTI_all_ values much more than climate variables (Table 7).

**Table 3:** Measures of phylogenetic diversity of Caatinga plants in each site (municipality, state) and different environment types (crystalline, sedimentary and inselberg): net relatedness index for all species (NRI_all_), woody species (NRI_wood_), and herbaceous species (NRI_herb_).

Sites	Environment type	NRI_all_	NRI_wood_	NRI_herb_
Crateús, CE	crystalline	**2.52**	**5.63**	-1.07
Floresta, PE	crystalline	-0.01	1.55	-1.17
Quixadá, CE	crystalline	**2.93**	**4.92**	-0.03
Floresta, PE	crystalline	1.39	**2.98**	-0.55
Floresta, PE	crystalline	1.20	**2.78**	-0.58
Crateús, CE	sedimentary	**3.46**	**5.31**	-0.66
Crateús, CE	sedimentary	**5.70**	**9.19**	-1.91
SJ Piauí, PI	sedimentary	**3.42**	**4.94**	**-2.07**
Quixadá, CE	inselberg	-0.59	1.07	-0.73
SJ Monte, PE	inselberg	**-4.19**	-0.05	**-3.58**
Agrestina, PE	inselberg	**-2.69**	0.92	**-2.96**
Esperança, PB	inselberg	**-3.34**	0.95	**-2.57**
F Santana, BA	inselberg	**-2.23**	-1.85	-0.12

Values in bold are those indicating significant clustering (> 1.96) or significant overdispersion (<- 1.96).

**Table 4:** Measures of phylogenetic diversity of Caatinga plants in sites (municipality, state) on different edaphic environments (crystalline, sedimentary, inselberg), NE Brazil: nearest taxon index for all species (NTI_all_), woody species (NTI_wood_), and herbaceous species (NTI_herb_).

Sites	Soil	NTI_all_	NTI_wood_	NTI_herb_
Crateús, CE	crystalline	**1.97**	**2.30**	1.63
Floresta, PE	crystalline	1.88	**3.51**	1.15
Quixadá, CE	crystalline	**2.78**	**3.03**	**2.96**
Floresta, PE	crystalline	1.76	**3.00**	1.15
Floresta, PE	crystalline	**2.07**	**2.94**	1.67
Crateús, CE	sedimentary	**2.39**	**3.44**	-0.01
Crateús, CE	sedimentary	1.73	**2.65**	1.12
SJ Piauí, PI	sedimentary	1.77	**3.51**	-1.19
Quixadá, CE	inselberg	-0.17	0.00	1.33
SJ Monte, PE	inselberg	-1.87	1.34	**-2.52**
Agrestina, PE	inselberg	-0.75	1.62	-1.10
Esperança, PB	inselberg	-0.98	1.75	-0.04
F Santana, BA	inselberg	-1.91	**-2.08**	0.72

Values in bold are those indicating significant clustering (> 1.96) or significant overdispersion (<- 1.96).

**Table 5:** Parameters of analyses of covariance (ANCOVAs) of the net relatedness index (NRI) among all species (NRI_all_), woody species (NRI_wood_), and herbaceous species (NRI_herb_), on different edaphic environments, by controlling the variation of climate variables.

Parameters	Net Relatedness Index					
	NRI_all_		NRI_wood_		NRI_herb_	
	coefficient	t (Pr>|t|)	coefficient	t (Pr>|t|)	coefficient	t (Pr>|t|)
Intercept	3.67	0.38 (0.72)	14.94	0.98 (0.36)	-10.08	-1.22 (0.27)
Annual Mean Temperature	0.10	0.32 (0.76)	-0.49	-1.01 (0.35)	0.38	1.43 (0.20)
Annual Mean Precipitation	-0.0039	-0.71 (0.50)	0.0023	0.27 (0.80)	-0.0013	-0.27 (0.79)
Precipitation of the Driest Quarter	0.0011	0.09 (0.93)	-0.035	-1.84 (0.12)	**0.023**	**2.22 (0.07)**
Crystalline	-1.89	-1.63 (0.15)	-0.14	-0.07 (0.94)	0.54	0.55 (0.60)
Inselberg	**-4.84**	**-3.82 (0.009)**	-2.42	-1.22 (0.27)	-0.82	-0.76 (0.48)
Spatial Filter	4.84	-1.38 (0.22)	-5.28	-0.96 (0.37)	-0.73	-0.24 (0.82)

Significant coefficient values at α = 0.10 are in bold. Numbers are presented to two decimal places or two significant digits.

**Table 6:** Parameters of analyses of covariance (ANCOVAs) of the nearest taxon index (NTI) for all species (NTI_all_), woody species (NTI_wood_), and herbaceous species (NTI_herb_), on different edaphic environments, by controlling the variation of climate variables.

Parameters	Nearest Taxon Index					
	NTI_all_		NTI_wood_		NTI_herb_	
	coefficient	t (Pr>|t|)	coefficient	t (Pr>|t|)	coefficient	t (Pr>|t|)
Intercept	1.47	0.29 (0.78)	5.90	1.31 (0.24)	-8.04	-0.72 (0.50)
Annual Mean Temperature	0.042	0.26 (0.80)	-0.20	-1.36 (0.22)	0.29	0.82 (0.44)
Annual Mean Precipitation	-0.00060	-0.21 (0.84)	0.0040	1.56 (0.17)	0.00039	0.06 (0.95)
Precipitation of the Driest Quarter	-0.012	-1.86 (0.11)	**-0.034**	**-6.11 (0.0009)**	0.0092	0.66 (0.53)
Crystalline	0.17	0.29 (0.78)	-0.27	-0.51 (0.63)	2.14	1.59 (0.17)
Inselberg	**-2.32**	**-3.55 (0.01)**	**-2.51**	**-4.28(0.005)**	0.39	0.27 (0.80)
Spatial Filter	-0.64	-0.35 (0.74)	**3.15**	**1.94 (0.10)**	-1.49	-0.37 (072)

Significant coefficient values at α = 0.10 are in bold. Numbers are presented to two decimal places or two significant digits.

**Table 7:** Partitioning of the variation explained solely by climate and edaphic environment for each phylogenetic diversity measure [net relatedness index for all species (NRI_all_), woody species (NRI_wood_), and herbaceous species (NRI_herb_); nearest taxon index for all species (NTI_all_), woody species (NTI_wood_), and herbaceous species (NTI_herb_)].

	climate	edaphic	interaction	adjusted R^2^
NRI_all_	-0.02	0.75	0.17	**0.91**
NRI_wood_	0.03	0.71	0.02	**0.76**
NRI_herb_	0.20	0.26	0.05	0.51
NTI_all_	0.02	0.64	0.25	**0.92**
NTI_wood_	0.28	0.40	0.25	**0.93**
NTI_herb_	-0.12	0.45	0.13	0.46

Adjusted R^2^ (Legendre and Legendre 2012, chapter 10) of the multiple regression of each phylogenetic diversity measure against climate variables and edaphic environment are shown. All values of adjusted R^2^ significantly different from zero (P<0.05) are in bold.

### Phylogenetic diversity for woody species

Patterns of phylogenetic diversity of woody species across edaphic environments corresponded roughly to those observed for all species (Tables 5 and 6, Fig. 2a,b and d,e). Lower values of NTI_wood_ were associated with inselberg environments and with increasing precipitation in driest quarter (Table 6). Similar associations were seen in NRI_wood_ but these differences were not statistically significant (Table 5). A strong phylogenetic clustering of woody species was evident in crystalline and sedimentary environments, with highly significant values of NRI_wood_ and NTI_wood_ for all but one of these sites whereas phylogenetic clustering was weaker or absent in inselbergs (Tables 3 and 4, Fig. 2b, e). Most of the variation in NRI_wood_ was explained by the edaphic environment while variation in NTI_wood_ was more evenly partitioned between climate and edaphic environment and their interaction (Table 7).

### Phylogenetic diversity for herbaceous species

The patterns of phylogenetic diversity observed for herbaceous species were quite different from those for woody species (Tables 5 and 6, Fig. 2c, f). Higher values of NRI_herb_ were significantly associated with increasing precipitation in driest quarter, but NTI_herb_ showed no association with the climate variables analyzed (Tables 5 and 6). Herbaceous species showed a marked tendency towards phylogenetic overdispersion in inselberg environments, with three inselberg sites yielding significantly negative values for NRI_herb_ (Table 3). A similar tendency to overdispersion was shown by negative values of NRI_herb_ for sedimentary and crystalline soils, but mostly not significant. In contrast there was a clear tendency towards phylogenetic clustering in crystalline soils for NTI_herb_ and no consistent pattern of either clustering or overdispersion in NTI_herb_ for sedimentary substrates or inselbergs (Table 4).

## Discussion

The composition and structure of plant communities is clearly driven by environmental factors. However, the spatial and temporal scale over which the structure of plant communities takes shape can vary depending on heterogeneity in environmental drivers. Moreover, the rate of ecological turnover and evolutionary adaptation of species within plant communities can also vary, depending on heterogeneity in life history.

Our study sought to explore the extent to which edaphic environment and climate structure phylogenetic pattern in woody and non woody components of the Caatinga flora. Our interest in including the often neglected herbaceous elements of the flora greatly constrained the number of studies which were suitable for inclusion in our analysis, with some 90% of site-based Caatinga surveys available in the literature omitting herbaceous species and/or Raunkiaerian life form data [17]. Nonetheless, our analyses revealed very clear and significant differences in phylogenetic pattern between Caatinga communities on different edaphic environments and between woody and herbaceous elements of the flora. As anticipated, we found significant differences in the extent of phylogenetic clustering of the plant community as a whole in different edaphic environments and these differences could be related to the relative water availability of those environments. However, phylogenetic clustering was most marked in communities in sedimentary environments which are associated with relatively greater water availability, but less nutrients in the soil. Phylogenetic clustering was less marked but still significant in communities in crystalline environments with intermediate water availability and least evident in inselberg communities in which water availability is most limited. We expected that a range of climatic factors that influence water availability might affect the degree of phylogenetic clustering observed but, of the climate parameters analyzed, only levels of precipitation of the driest quarter had a significant effect. Importantly, that effect was not significant when the flora was considered as a whole but only when woody and herbaceous components were considered separately. In fact, the phylogenetic clustering which is demonstrably a feature of the woody component of Caatinga communities is much less prevalent in the herbaceous component. Although our study did not focus explicitly on effects at different spatial scales, the use of two measures of phylogenetic diversity, distinguishing between phylogenetic clustering at the terminals of the phylogeny (NTI) and effects involving deeper nodes of the tree (NRI) allows us to explore our results in terms of different temporal scales.

Among the environmental variables, climatic variables are likely to show shorter term change than edaphic environment. Moreover, communities of herbaceous species, most of them corresponding to therophytes in the Caatinga [13,51], are likely to undergo changes in species composition on a shorter time-scale than woody plants because they are shorter lived. The two measures of phylogenetic diversity we used have a similar temporal dichotomy: NRI encompasses long-term evolution whereas NTI is weighted towards more recent adaptation. Edaphic environment (different soil substrate types) is a major driver of long-term patterns of plant association, reflected in NRI and the pattern associated with woody plants, while climate appears to be the more important driver of NTI and herbaceous plant distributions. More specifically, comparison of Tables 5 & 6 shows the impact of both ecological and evolutionary time-scales against different edaphic conditions (rupicolous environment in inselbergs) and climate (precipitation of the driest quarter). Phylogenetic overdispersion, or a reduced level of phylogenetic clustering, is associated with the inselberg edaphic environment (long term factor). Phylogenetic clustering is associated with precipitation of the driest quarter (short term factor). These effects are evident in Table 7, with a clear correspondence of long term drivers with NRI (long term phylogenetic response), and vice versa. Fig. 2 shows a similar difference between NRI and NTI and between the herbaceous and woody components, with the former showing a pattern of phylogenetic overdispersion for herbaceous species in contrast to an overall tendency towards phylogenetic clustering.

Our results also clearly demonstrate the importance of water availability as a driver of plant associations in SDTFs. In the case of Caatinga, inselberg substrate and low rainfall in the dry season strongly limit water availability, resulting in different patterns of diversity in sites showing these conditions. The important point here is that SDTF vegetation across the Caatinga is very heterogeneous and responding on a very local scale to microclimate and microenvironment.

The Caatinga flora is thought to have two origins. During the upper Tertiary, large denudational events resulted in a generalized pediplanation in the Northeastern region of Brazil [58,59]. The widespread crystalline lowlands that occupy most of the area within Caatinga resulted from this process. With the pediplanation, sedimentary basins were formed and remained as disjunct sites within the crystalline landscapes (Fig. 1). In a large review of the Leguminosae, Queiroz [12,60] has identified many taxa endemic to these disjunct sedimentary landscapes. He hypothesized that the original, endemic flora of the Caatinga was that present in the sedimentary landscapes, while the flora of crystalline sites was, to a great extent, a result of migration of taxa from other SDTF in the continent, which arrived and mixed with the original flora [12,60].

Ecological theory suggests contrasting conditions that promote either phylogenetic clustering or phylogenetic overdispersion [21,61] based on the interaction between rates of trait evolution and environmental filtering versus competitive interactions. We propose that such effects may underlie patterns of phylogenetic diversity in the Caatinga. For example, if the flora of sedimentary landscapes is higher in endemism and more locally evolved, while the crystalline flora is mostly migrant [12], we would expect the phylogenetic structure of these communities to differ clearly and to find a more heterogeneous structure in the crystalline. Moreover, the geographically distributed nature of inselberg habitats means that they are in effect islands potentially subject to even higher levels of migration and consequent higher species turnover than crystalline sites. This gradient in historical species composition was clearly reflected in NRI, which showed strong phylogenetic clustering in sedimentary sites, weaker phylogenetic clustering in crystalline sites, and phylogenetic overdispersion in inselberg sites. On the other hand, NTI for sedimentary and crystalline showed similar phylogenetic clustering for nearer neighbor (congeneric/confamiliar) taxa, with inseberg sites again showing phylogenetic overdispersion (Fig. 2). This might reflect stronger recent environmental niche conservatism on these more widespread habitat types.

Thus, our results show that environmental filtering is an important driver of phylogenetic diversity in the Caatinga, perhaps acting at different spatial scales or in different habitat types. However, we have not investigated competitive exclusion as a driver of phylogenetic diversity in this system, so we cannot be conclusive about the relative importance of these drivers, but our findings could be a useful foundation for development of more targeted ecological investigation of these processes.

## Conclusion

Our findings provide clear evidence of complex structuring of Caatinga communities over differing spatial and temporal scales, with different elements of the flora responding to common drivers in different ways. This study clearly demonstrated the impact of interaction between spatial and temporal heterogeneity in environmental drivers in shaping patterns of species diversity across a heterogeneous landscape. This finding is of potential significance to understanding patterns of biodiversity in SDTFs because this is a biome that is typically characterized by extreme environmental heterogeneity and limited dispersal [9], and it also helps us to understand what distinguishes this biome from other biomes characterized by lower levels of β-diversity.

With regard to the Brazilian Caatinga, during most of the 20^th^ century, this region was assumed to be species poor due to its harsh, semiarid climate. However, recent floristic and phytogeographical studies [12,13,15–17] are revealing that Caatinga has a high floristic biodiversity with distinctive flora associated with sedimentary, crystalline, and inselberg terrains. This reinforces the need to protect a representative proportion of the environments existing within Caatinga as different plant communities as well as different lineages seem to be represented in each environment type. Caatinga is one of the least protected natural ecoregions of Brazil, with roughly half of its area already degraded by humans and less than 2% of its area within Integrally Protected Natural Reserves (Unidades de Conservação de Proteção Integral) [62]. As we have shown here, not only species, but also lineages (and the functional attributes related to them, represented by life-forms) differ among the different environment types. Any program to establish new nature reserves must take into account the necessity to protect a proportion of each of these three environment types (sedimentary, crystalline and inselberg) within Caatinga in order to guarantee survival of species, communities and lineages.

## Supporting Information

S1 Dataset Estimates of MPD and MNTD accompanied by the standardized effect sizes (SES) for the whole plant community and for woody or herbaceous plants across sample sites in the Caatinga Phytogeographical Domain. (TXT)
